# Evaluation of the available cholesterol concentration in the inner leaflet of the plasma membrane of mammalian cells

**DOI:** 10.1016/j.jlr.2021.100084

**Published:** 2021-05-05

**Authors:** Pawanthi Buwaneka, Arthur Ralko, Shu-Lin Liu, Wonhwa Cho

**Affiliations:** Department of Chemistry, University of Illinois at Chicago, Chicago, IL, USA

**Keywords:** cholesterol, plasma membrane, inner leaflet, transbilayer asymmetry, cholesterol availability, ratiometric sensors, quantitative fluorescence imaging, perfringolysin O toxin, Osh4, cholesterol pools, 25HC, 25-hydroxycholesterol, EGFP, enhanced green fluorescence protein, FP, fluorescence protein, GUV, giant unilamellar vesicle, HLF, human lung fibroblast, HPAEC, human pulmonary artery endothelial cell, HUVEC, human umbilical vein endothelial cell, IPM, inner leaflet of the plasma membrane, LUV, large unilamellar vesicle, MEF, mouse embryonic fibroblast, OPM, outer leaflet of the plasma membrane, PFO, perfringolysin O, PI(4)P, phosphatidylinositol-4-phosphate, PI, phosphatidylinositol, PIP_2_, phosphoinositol-4,5-bisphosphate, PM, plasma membrane, POPC, 1-palmitoyl-2-oleoyl-*sn*-glycero-3-phosphocholine, POPE, 1-palmitoyl-2-oleoyl-*sn*-glycero-3-phosphoethano8lamine, POPS, 1-palmitoyl-2-oleoyl-*sn*-glycero-3-phosphoserine, SAG, 1-stearoyl-2-arachidonoyl-*sn*-glycerol, SAPC, 1-stearoyl-2-arachidonoyl-*sn*-glycero-3-phosphocholine, SPR, surface plasmon resonance

## Abstract

Cholesterol is an essential component of the mammalian plasma membrane involved in diverse cellular processes. Our recent quantitative imaging analysis using ratiometric cholesterol sensors showed that the available cholesterol concentration in the inner leaflet of the plasma membrane (IPM) is low in unstimulated cells and increased in a stimulus-specific manner to trigger cell signaling events. However, the transbilayer distribution of cholesterol in the plasma membrane of mammalian cells remains controversial. Here we report a systematic and rigorous evaluation of basal IPM cholesterol levels in a wide range of mammalian cells with different properties employing cholesterol sensors derived from the D4 domain of the Perfringolysin O toxin and a sterol-transfer protein, Osh4. Results consistently showed that, although basal IPM cholesterol levels vary significantly among cells, they remain significantly lower than cholesterol levels in the outer leaflets. We found that IPM cholesterol levels were particularly low in all tested primary cells. These results support the universality of the low basal IPM cholesterol concentration under physiological conditions. We also report here the presence of sequestered IPM cholesterol pools, which may become available to cytosolic proteins under certain physiological conditions. We hypothesize that these pools may partly account for the low basal level of available IPM cholesterol. In conclusion, we provide new experimental data that confirm the asymmetric transbilayer distribution of the plasma membrane cholesterol, which may contribute to regulation of various cellular signaling processes at the plasma membrane.

Cholesterol is a major and essential component of the mammalian plasma membrane (PM) (1, 2, 3, 4). Cholesterol maintains the biophysical properties of the PM (1, 2), including rigidity and permeability (5), and serves as a precursor for steroids (6) and bile acids (7). It is also implicated in the formation of membrane microdomains, including lipid rafts, which may compartmentalize various cell signaling events (8). Recently, cholesterol has been shown to interact with a wide variety of cellular proteins (9). Multiple studies have reported that cholesterol regulates the structure and function of various integral membrane proteins (10), including ion channels (11, 12) and G protein-coupled receptors (13). Our recent studies have shown that cholesterol at the inner leaflet of the PM (IPM) specifically interacts with cytosolic proteins that coordinate diverse cell signaling events (14, 15, 16). These results suggest that cholesterol might function as a site-specific signaling lipid, just as phosphoinositides (17) and diacylglycerol (18). To test this notion, we performed simultaneous in situ quantitative imaging of available cholesterol in the two leaflets of the PM of mammalian cells using orthogonal ratiometric sensors (19). The results showed asymmetric distribution of cholesterol across the PM, with its available concentration at the IPM ([Chol]_*i*_) lower than that at the outer leaflet (OPM) ([Chol]_*o*_) by an order of magnitude (19). This asymmetry is maintained by the ATP-dependent cholesterol floppase activity of ABCA1 and ABCG1 and the ability of sphingomyelin at OPM to deter reverse translocation of cholesterol to IPM (19). Furthermore, [Chol]_*i*_ was increased specifically in response to canonical Wnt ligands in a dose- and time-dependent manner, leading to activation of canonical Wnt signaling that facilitates cell growth and proliferation (19). Our quantitative cholesterol imaging also provided evidence for the notion that Patched1 serves as a tunable cholesterol floppase that controls the hedgehog signaling activity by modulating [Chol]_*i*_ (20).

The transbilayer distribution of cholesterol in the PM has remained controversial (21, 22). Specifically, the low [Chol]_*i*_ values determined by our method (19) are in disagreement with other reports indicating that [Chol]_*i*_ is higher than [Chol]_*o*_ (23, 24). Also, our results are apparently at odds with the previous reports indicating that the total cholesterol concentration in the PM is about 40 mol% (25, 26, 27), because the average cholesterol concentration calculated from our [Chol]_*i*_ and [Chol]_*o*_ values is ≈22 mol% (19). In light of growing interest in cholesterol-mediated cell signaling and regulation in health and disease (28, 29, 30), it is imperative that this controversy should be resolved for the cholesterol research community. The discrepancy may derive from the fact that previous studies mostly relied on a single analytical method (e.g., mass spectrometry, fluorescence, and radioactivity) and reagent (e.g., various cholesterol probes and fluorescent cholesterol) on a single or a limited number of cell type(s) (22). A systematic and rigorous quantitative analysis of [Chol]_*i*_ and [Chol]_*o*_ employing multiple techniques and probes on diverse cell types has not been reported. Also, the PM cholesterol concentration has not been quantified in primary cells. In this study, we systematically and rigorously determined the [Chol]_*i*_ (and [Chol]_*o*_) values in a wide variety of cell types, including primary fibroblasts and epithelial and endothelial cells, by three independent methods using different cholesterol sensors. We also investigated a new mechanism by which availability of IPM cholesterol is controlled under physiological conditions.

## Materials and methods

### Materials

All cell lines including human pulmonary artery endothelial cells (HPAECs), human lung fibroblasts (HLFs), and human umbilical vein endothelial cells (HUVECs) were purchased from ATCC. 1-Palmitoyl-2-oleoyl-*sn*-glycero-3-phosphocholine (POPC), 1-palmitoyl-2-oleoyl-*sn*-glycero-3-phosphoethanolamine (POPE), 1-palmitoyl-2-oleoyl-*sn*-glycero-3-phosphoserine (POPS), 1-stearoyl-2-arachidonoyl-*sn*-glycero-3-phosphocholine (SAPC), 1-stearoyl-2-arachidonoyl-*sn*-glycerol (SAG), cholesterol, 25-hydroxycholesterol (25HC), and soy L-α-phosphatidylinositol (PI) were purchased from Avanti Polar Lipids.1,2-Dipalmitoyl derivatives of PIP_2_ and PI(4)P were from Cayman Chemical Co. (Cat no. 10008115). Fibronectin for the cell culture plate coating was purchased from Millipore Sigma (Cat no. 341631, lot no. 3273650). Fibroblast basal medium and fibroblast growth kit-low serum for HLF cells were purchased from ATCC. A transfection reagent JetPRIME was from Polyplus transfection. Mitochondria marker MitoTracker™ Deep Red FM was purchased from Invitrogen. Human and mouse Cav1 siRNAs were purchased from Qiagen and Integrated DNA Technologies, respectively.

### Preparation of WCR-YDA

All constructs of D4 WT, D434A, and YDA were subcloned into the pET-30a vector with an N-terminal His_6_-tag, and proteins were expressed in *E. coli* BL21 RIL codon plus cells (Stratagene) and purified using the Ni-NTA agarose affinity resin (Marvelgent) as described previously (19). For preparation of WCR-YDA, the YDA-bound resin was resuspended with WCR (1:10 molar ratio) in 5 ml of labeling buffer [50 mM Tris, pH 8.05, containing 150 mM NaCl, 20 mM imidazole, 50 mM arginine, 50 mM glutamate, and 1 mM Tris(2-carboxyethyl)phosphine (TCEP)] and the mixture was gently shaken for 2 h at room temperature, or at 4°C overnight in a gyratory shaker. WCR-YDA was then washed with 50 ml of the wash buffer (80 mM Tris, pH 7.9, 300 mM NaCl, 40 mM imidazole) containing 4% (v/v) dimethyl sulfoxide and then with 300 ml of the wash buffer. WCR-YDA was eluted from the resin with the elution buffer (50 mM Tris, pH 7.9, 300 mM NaCl, 300 mM imidazole). Collected fractions were concentrated in an Amicon Ultra 0.5 ml Centrifugal Filter (Millipore), and the buffer solution was exchanged to 20 mM Tris, pH 7.4, 160 mM NaCl. The protein concentration of the WCR-YDA solution was determined by the Bradford assay. All steps were performed at 4°C unless otherwise mentioned.

### Preparation of WCR-eOsh4

The Osh4 gene was a generous gift from Dr James Hurley. After removing the N-terminal 40 residues from Osh4, the truncated gene was cloned into the pET 30a vector with N- and C-terminal His_6_-tags. All endogenous cysteines (C68, C98, and C229) were mutated to Ser and the K108C mutation was introduced as a single fluorophore labeling site in the membrane-binding region. The K109A mutation was introduced to decrease the affinity of the protein for 25HC, yielding *e*Osh4 (Osh4-△^1-40(C68S/C98S/K108C/K109A/C229S)^). eOsh4 was transformed to *E. coli* BL21 RIL codon plus cells (Stratagene) for bacterial expression. A preculture was prepared from a single colony in 10 ml of Luria-Bertani medium with 50 μg/ml kanamycin and was incubated in a shaker overnight at 37°C or until it got cloudy. Ten milliliters of preculture was transferred to 1 l of main culture with 50 μg/ml kanamycin and incubated in a shaker at 37°C until *A*_600_ reached 0.6. Then protein expression was induced at 18°C with 0.5 mM isopropyl β-d-1-thiogalactopyranoside for 16 h. The induced culture was aliquoted to 250 ml and centrifuged at 4,000 *g* for 10 min. Cell pellets were stored at −80°C until use. The cell pellets were resuspended with 20 ml of the lysis buffer (50 mM Tris HCl [pH 7.9], 300 mM NaCl, 10 mM imidazole, 10% glycerol, 1 mM phenylmethanesulfonylfluoride, and 1 mM dithiothreitol) and lysed by sonication. The lysate was centrifuged at 44,000 *g* for 30 min and the supernatant was mixed with 1 ml of Ni NTA agarose resin (Marvelgent Biosciences Inc.) and incubated at 4°C for 2 h with gentle shaking. For preparation of WCR-*e*Osh4, the *e*Osh4-bound resin was treated with WCR as described for WCR-YDA.

### Surface plasmon resonance analysis

All surface plasmon resonance (SPR) measurements were performed at 23°C in 20 mM Tris, pH 7.4, containing 0.16 M NaCl using a lipid-coated L1 chip in the BIACORE X-100 system (GE Healthcare) as described previously (31). IPM-mimetic LUVs with varying lipid compositions and POPC (or POPC/POPS (8:2)) vesicles were used as the active surface and the control surface, respectively. Sensorgrams were obtained for both association and dissociation phases but only the association phases were used for data analysis. Equilibrium measurements were performed at a flow rate of 10 μl/min to allow the resonance unit values for the association phase to reach near equilibrium (*R*_*eq*_). Each sensorgram was background-corrected by subtracting the control surface response from the active surface response. For calculation of apparent dissociation constant (*K*_d_), a minimum of five different protein concentrations (*P*_o_) were injected to collect a set of *R*_*eq*_ values that were plotted against *P*_o_. *K*_d_ was then determined by nonlinear least-squares analysis of the binding isotherm using the equation *R*_*eq*_ = *R*_*max*_/(1 + *K*_d_/*P*_o_), where *R*_*max*_ indicates the maximal *R*_*eq*_ value (see Fig. 3D). Since the concentration of lipids coated on the sensor chip cannot be accurately determined, *K*_d_ is defined as *P*_o_ yielding half-maximal binding with a fixed lipid concentration. Each measurement was repeated at least three times to determine average and SD values.

### Cell culture maintenance and preparation for imaging

All cells were seeded into 8-well chamber slides (Lab-Tek, Thermo Fisher Scientific) or 55 mm round glass-bottom plates and grown at 37°C in a humidified atmosphere of 95% air and 5% CO_2_. HeLa WT and ABCA1-KO HeLa cells, HEK293 cells, and mouse embryonic fibroblast (MEF) cells were grown in phenol red-free Dulbecco's modified Eagle medium (DMEM) (Life technologies) supplemented with 10% (v/v) fetal bovine serum (FBS) (Sigma-Aldrich) and 100 U/ml penicillin-streptomycin solution (Life technologies). MA10 cells were seeded into fibronectin (10 μg/ml)-coated 8-well chamber slides and grown in the DMEM/Ham's F12 (1:1) mixed medium (Life technologies) supplemented with 15% (v/v) horse serum (Sigma-Aldrich). HLF cells were grown in the fibroblast basal medium (ATCC) supplemented with the fibroblast growth kit-low serum (ATCC). HUVECs and HPAECs were maintained in the ECGM2 medium supplemented with 10% FBS and growth factor mix (PromoCELL). HPAECs were plated onto 55 mm plates treated with 0.25% gelatin and 10 μg/ml fibronectin. Quality of cells was maintained by passaging each cell line according to the ATCC guidelines. Dead cells and cell debris were washed with Dulbecco's phosphate-buffered saline before every passage. Some dead cells resulting from transfection were also removed from the plates by washing them with Dulbecco's phosphate-buffered saline three times before imaging.

### Preparation of HeLa cell extract

HeLa cells were grown in two to three 10 cm culture plates as described above until the plates were fully confluent. The growth medium was removed from the plates and cells were washed with ice-cold phosphate buffer saline three times. Then the cells in each plate were lysed with 1 ml of the reporter lysis buffer (Promega) with the protease inhibitor cocktail (RPI) according to the manufacturer's protocol. The cell lysate was centrifuged at 15,000 rpm at 4°C for 10 min to remove cell debris, and the supernatant was ultracentrifuged (Beckman Coulter optima XE-100) at 100,000 *g* for 1 h at 4°C to remove nuclei, cellular organelles, and membrane fractions. The supernatant was carefully isolated and concentrated with Amicon Ultra 0.5 ml Centrifugal Filter 3K (Millipore). The total protein concentration of the extract was determined by the Bradford assay.

### siRNA transfection of mammalian cells

Cav1 knockdown was performed by human and mouse Cav1 siRNA, respectively, as reported previously (32). HPAECs and HEK 293 cells were grown to 60% confluency and transfected with human Cav1 siRNA using the JetPRIME system (Polyplus-transfection) according to the manufacturer's protocol. After cells were incubated with siRNA for 3 days, they were split and plated onto 55 mm dishes. Similarly, MEF cells were treated with mouse siRNA for 3 days.

### Correlation between EGFP concentration and fluorescence intensity

To estimate the cellular concentration of EGFP from its fluorescence intensity, we performed calibration using EGFP solutions with defined concentrations. After purifying bacterially expressed EGFP with a C-terminal His_6_ tag using the Ni-NTA agarose affinity resin, EGFP in the phosphate buffer saline solution (pH 7.4) was concentrated in an Amicon Ultra 0.5 ml Centrifugal Filter (Millipore) and its final concentration determined by the Bradford assay. After serial dilution, EGFP solutions with varying concentrations were added to the 8-well chamber slides and their images were collected with a custom-modified six channel FV3000 (Olympus) confocal laser scanning microscope with the same setting employed for cell imaging. Collected images were imported to and analyzed in Image-Pro Plus 7 (Media Cybernetics). From the total intensity of each well containing different concentrations of EGFP, mean optical density (*I*_*av*_) (counts/pixel) values were calculated and plotted against the EGFP concentration to yield the calibration plot (see supplemental Fig. S3).

### Subcellular localization analysis of EGFP-D4 domains

Two different expression vectors were used for the high and low expression of EGFP-D4 WT and mutants in mammalian cell lines. For high expression the pEGFP C1 vector was used. Low-expression vectors were generated by removing the CMV enhancer and truncating the CMV promotor of pEGFPC1 vector by *AatII*. Digested vectors were re-ligated with T4 DNA ligase, and genes for the D4 WT or mutants were cloned into them using *XhoI* and *BamHI* restriction sites. EGFP-D4 constructs in the high- or low-expression vector were transiently transfected to cells in the phenol red-free DMEM medium with the JetPRIME system. For transfection of HLF cells, EGFP-D4 constructs were cloned into the pLego iV2 plasmid. To generate lentivirus, HEK 293T cells were cotransfected with target vector, psPAX and PMD2G, with the JetPRIME system. After replacing the medium after 6 h, cells were grown for 60 h. The viral solution was collected by filtering the cell medium with a 0.45 μm filter. HLF cells plated in 8-well chamber slides were infected with the virus in the presence of 10 μg/ml polybrene (Sigma-Aldrich). Infected cells were incubated at 37°C for 24 h, and the medium was exchanged with the complete growth medium. After transfection, 70–80% of the cells displayed fluorescence. The same number (2.5 × 10^4^) of cells were plated on 8-well chamber slides for microscopic imaging. Images were obtained with the custom-designed six channel Olympus FV3000 confocal microscope with the environmentally controlled full enclosure incubator (CellVivo) 4–6 h after transfection for cell lines and 72 h after infection for HLF cells. Cells were maintained at 37°C and with 5% CO_2_ atmosphere throughout the imaging period to maintain the cell viability. Collected images were imported to and analyzed in Image-Pro Plus 7. The protein expression level of each cell was estimated from its *I*_*av*_ using the calibration plot of EGFP (see supplemental Fig. S3). For the high protein expression cell groups, only those cells with *I*_*av*_ ≥ 750 (arbitrary unit/pixel) (i.e., [protein] ≥500 nM) were selected for further analysis. For the low-expression cell groups, cells with *I*_*av*_ ≤ 400 (i.e., [protein] ≤250 nM) were selected for imaging. Likewise, only those HLF cells with *I*_*av*_ ≥ 750 and with *I*_*av*_ ≤ 400 were selected for further analysis as high- and low-expression cells, respectively. The degree of IPM localization (*I*_PM_/*I*_Cytosol_ = the ratio of fluorescence intensity at PM to that in the cytosol) of EGFP-D4 domains for each selected cell was calculated by the intensity line profile analysis in Image-Pro Plus7. Briefly, at least five different lines were drawn across the cross-sectional image of each cell and the average *I*_PM_ and *I*_Cytosol_ values were calculated along the lines. Typically, >20 cell images were analyzed for each data set to determine the average and SD values.

### Calibration of cholesterol sensors

In vitro calibration of WCR-YDA and WCR-*e*Osh4 was performed with IPM-mimetic giant unilamellar vesicles (GUVs) (POPC/POPE/POPS/PI/cholesterol/PIP_2_ (20/50-x/20/9/x/1: *x* = 0–20 mol%)) as described previously with minor modifications (19). These GUVs were mixed with WCR-YDA or WCR-*e*Osh4 (200 nM), and fluorescence imaging was performed with the custom-designed six channel Olympus FV3000 confocal microscope with the environmentally controlled full enclosure incubator (CellVivo) (see supplemental Fig. S2). WCR-YDA (or WCR-*e*Osh4) was excited with the 488 nm laser source, and the emission intensity was collected in two separate channels with the spectral detector setting of 540–620 nm (orange channel) and 630–660 nm (red channel). For each cholesterol concentration, at least 10 GUVs were selected for image analysis by Image-Pro Plus7. Calibration curve fitting for WCR-YDA was performed by nonlinear least-squares analysis using the equation *F*_O_/*F*_R_ = (*F*_O_/*F*_R_)_min_ + ((*F*_O_/*F*_R_)_max_ − (*F*_O_/*F*_R_)_min_)/(1 + Exp((*K*_1/2_ − [Chol])/*S*)). *F*_O_/*F*_R_, *K*_d_, (*F*_O_/*F*_R_)_max_, (*F*_O_/*F*_R_)_min_, and *S* are the ratio of the fluorescence intensity in the orange channel to that in the red channel, equilibrium dissociation constant (in mol%), the maximal and minimal *F*_O_/*F*_R_ values, and the Slope (or Stiffness) constant, respectively (19). For WCR-*e*Osh4 with simple hyperbolic binding curves, data were fit using a Langmuir-type binding equation: *F*_O_/*F*_R_ = (*F*_O_/*F*_R_)_min_ + ((*F*_O_/*F*_R_)_max_ − (*F*_O_/*F*_R_)_min_)/(1 + *K*_1/2_[Chol]). In vitro calibration of DAN-D4 was performed as described (19).

### In situ quantitative imaging

The same number (2.5 × 10^4^) of cells were seeded into 100 mm round glass-bottom plates (MatTek) and grown at 37°C in a humidified atmosphere of 5% CO_2_ in phenol red-free DMEM supplemented with 10% (v/v) FBS, 100 U/ml penicillin G, and 100 mg/ml streptomycin sulfate and cultured in the plates for about 24 h before lipid quantification. Imaging was performed with the custom-designed six channel Olympus FV3000 confocal microscope with the environmentally controlled full enclosure incubator (CellVivo). Cells were maintained at 37°C and with 5% CO_2_ atmosphere throughout the imaging period to maintain the cell viability. Typically, 20–30 fl of the sensor solution was microinjected into the cell to reach the final cellular concentration of 200–400 nM. All image acquisition and imaging data analysis were performed as described for GUV calibration. All [Chol]_*i*_ and [Chol]_*o*_ determination was performed using the GUV calibration curves as described previously (19). The three-dimensional display of the local lipid concentration profile was calculated using the Surf function in MATLAB. *P* values were calculated by the Student's *t*-tests.

## Results

### Membrane binding properties of D4 domain-derived IPM cholesterol sensors

**Fig. 1:**
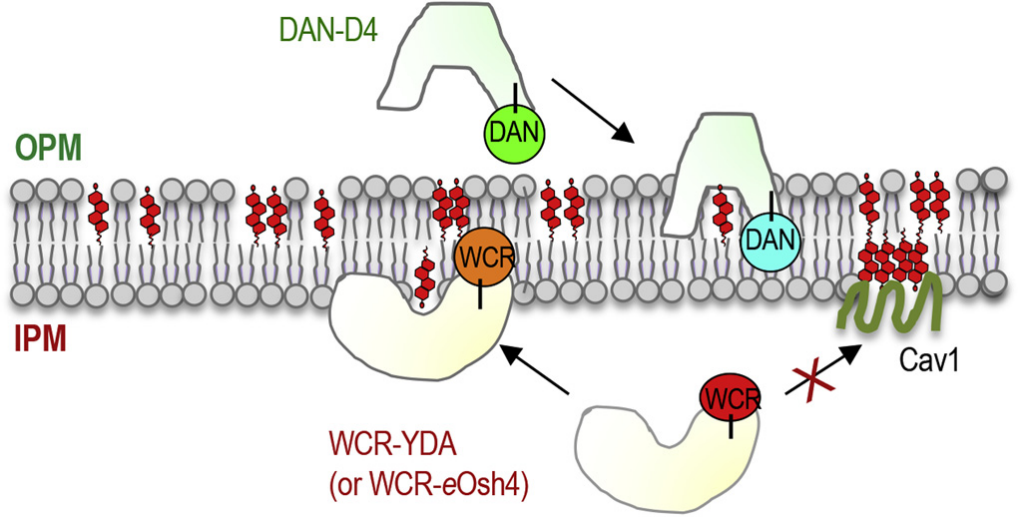
A general strategy to quantify IPM and OPM cholesterol. For simultaneous ratiometric quantification DAN-D4 (showing a green to blue shift when bound to the membrane) was added to the medium, whereas WCR-YDA (or WCR-*e*Osh4) (undergoing a red to orange shift) was microinjected into the cell. [Chol]_*i*_ and [Chol]_*o*_ were simultaneously calculated from fluorescence signals from four separate detectors through ratiometric calibration. Some IPM cholesterol molecules are sequestered by membrane proteins, such as Cav1, and thus unavailable to cytosolic cholesterol sensors and signaling proteins.

*Perfringolysin* O (PFO) toxin is a cholesterol-specific cytolysin (33, 34, 35). Its cholesterol-binding, noncytotoxic D4 domain and mutants with improved membrane affinity have been commonly used as a cholesterol probe, typically as a fluorescence protein (FP)-fusion protein (34, 36, 37, 38). Earlier studies have shown that PFO and D4 domain proteins exhibit unique biphasic cholesterol concentration dependence, in which their interaction with cholesterol-containing vesicles is abruptly increased above a threshold cholesterol concentration (35, 37, 39, 40, 41). This phenomenon has been often attributed to the formation of stoichiometric complexes between cholesterol and phospholipids with appropriate acyl chain length and saturation, sphingolipids in particular, through weak interactions (42, 43). That is, below a threshold concentration all cholesterol molecules are complexed with phospholipids and thus have low chemical activity and are not available for interaction with soluble proteins, whereas uncomplexed (or excess) cholesterol existing above the threshold value is more available for interaction with these proteins (39). For instance, the wild-type (WT) D4 domain does not show significant binding until the cholesterol content in the membrane reaches 40 mol%. Also, cholesterol binding of the WT D4 domain depends greatly on the lipid environment of cholesterol-containing membranes. In practical terms, the sharp cholesterol dependence of the D4 domain severely restricts its linear response range and its environment sensitivity may limit its utility as a cholesterol-specific sensor. To overcome these major limitations, we previously prepared a panel of D4 mutants with variable cholesterol affinity, including YDA (Y415A/D434W/A463W) whose linear response range reaches below 1 mol% cholesterol (19). We then prepared ratiometric cholesterol sensors by chemically conjugating these D4 proteins with an environment-sensitive amphiphilic fluorophore. For instance, our original IPM cholesterol sensor, NR3-YDA, was prepared by labeling YDA with NR3 (44) and the OPM cholesterol sensor by labeling WT D4 with acrylodan (DAN-D4) (Fig. 1).

**Fig. 2:**
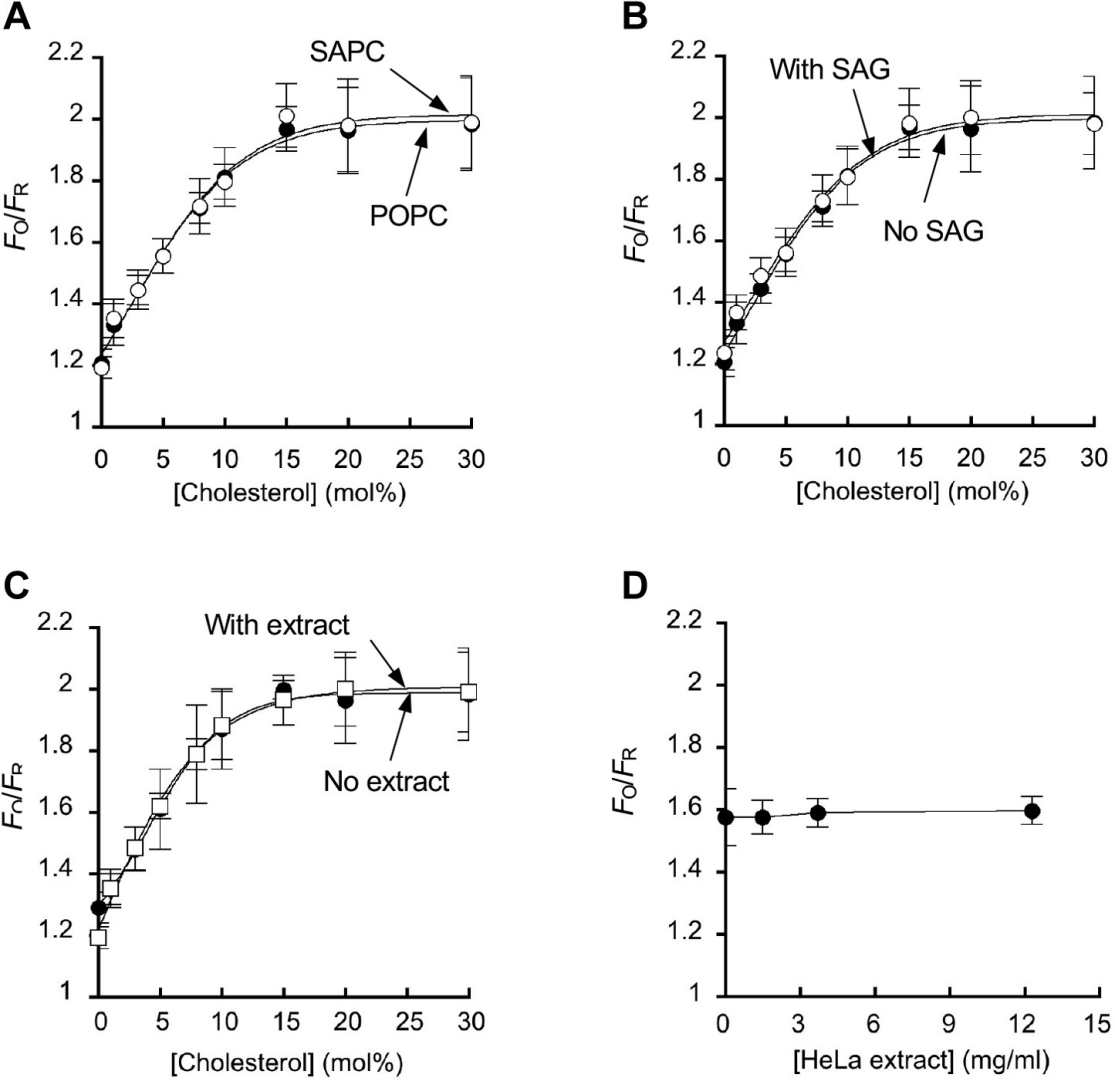
Effects of lipids and proteins on membrane binding of WCR-YDA. A: Effect of the acyl chain structure of phospholipids on cholesterol-dependent vesicle binding of WCR-YDA. Lipid compositions of GUVs were POPC/POPE/POPS/PI/cholesterol/PIP_2_ (20/50-x/20/9/x/1: *x* = 0–30 mol%) (open circles) and SAPC/POPE/POPS/ PI/cholesterol/PIP_2_ (20/40-x/30/9/x/1: *x* = 0–30 mol%) (closed circles). B: Effect of the lipid composition on cholesterol-dependent GUV binding of WCR-YDA. Lipid compositions of GUVs were POPC/POPE/POPS/PI/cholesterol/PIP_2_ (20/50-x/20/9/x/1: *x* = 0–30 mol%) (closed circles) and POPC/POPE/POPS/PI/cholesterol/PIP_2_/ SAG (19/40-x/30/9/x/1/1: *x* = 0–30 mol%) (open circles). C: Effect of the HeLa cell extract on cholesterol-dependent GUV binding of WCR-YDA. The lipid composition of GUVs was POPC/POPE/POPS/PI/cholesterol/PIP_2_ (20/50-x/20/9/x/1: *x* = 0–30 mol%). The cell extract from HeLa cells (total protein concentration = 2 mg/ml) was added to the mixture. D: Effect of varying concentrations of the HeLa cell extract on GUV binding of WCR-YDA. The lipid composition of GUVs was POPC/POPE/POPS/PI/cholesterol/PIP_2_ (20/45/20/9/5/1), and the total protein concentration of HeLa cell extract was varied from 0 to 12 mg/ml. GUV binding of WCR-YDA was measured by confocal microscopy. *F*_O_ and *F*_R_ indicate orange and red channel fluorescence intensity, respectively. The orange channel depicts membrane-bound sensors, whereas the red channel shows membrane-bound plus free sensors. Cholesterol dependency plots were analyzed by nonlinear least-squares fit using the equation *y* = *y*_min_ + (*y*_max_ - *y*_min_)/(1 + Exp((*K*_1/2_ - [Chol])/*S*)) yielded *K*_1/2_, *y*_max_, *y*_min_, and *S* values (19) and the theoretical curves were constructed using these parameters. *K*_1/2_, *y*_max_, *y*_min_, and *S* are [Chol] yielding half maximal binding (in mol%), the maximal and minimal *y* values and the Slope (or Stiffness) constant. Each data point is the average ± SD from >3 independent measurements.

To improve on the IPM cholesterol sensor, we developed a newer version, WCR-YDA. WCR is a newly developed environment-sensitive fluorophore that replaces NR3 because of its significantly improved spectral properties over NR3, allowing more accurate lipid quantification (20, 45). To demonstrate that WCR-YDA can accurately and specifically quantify [Chol]_*i*_, we thoroughly measured the effects of the lipid environment and cytosolic proteins on its membrane binding properties. We first measured the effect of the lipid environment of IPM-mimetic GUVs on the membrane-binding properties of WCR-YDA by fluorescence microscopy supplemental Fig. S2. Specifically, we determined the effects of two factors that have been reported to affect the cholesterol availability in the membrane, the acyl group of phospholipids (46) and the presence of membrane-disrupting molecules (47), under physiologically relevant conditions. To assess the effect of acyl groups, we measured the binding of WCR-YDA to IPM-mimetic GUVs containing POPC and SAPC, respectively. SAPC has three more *cis*-double bonds in the *sn*-2 acyl chain than POPC and is thus expected to have a more dramatic effect on the physical properties of the membrane, including membrane packing and cholesterol-phospholipid interaction (48). As shown in Fig. 2A, WCR-YDA did not distinguish between POPC and SAPC, in contrast to D4-WT that bound large unilamellar vesicles (LUVs) containing SAPC better than those containing POPC (supplemental Fig. S1). Of importance, the linear response range of WCR-YDA is much broader (i.e., 0–15 mol%) than unlabeled WT (supplemental Fig. S1) and mutant D4 domains (35, 37, 40, 41) because of its greatly attenuated cholesterol dependency. The same trend was reported for NR3-YDA (19), underscoring the favorable effect of mutations in YDA and the conjugated amphiphilic fluorophores (i.e., NR3 and WCR) on the membrane binding properties of the sensor. We also examined the effect of diacylglycerol on cholesterol binding of WCR-YDA. It was reported that diacylglycerol makes cholesterol more available by displacing it from phospholipids (47). SAG is the primary diacylglycerol in the IPM that is produced from 1-stearoyl-2-arachidonoyl-*sn*-glycero-3-phosphoinositol-4,5-bisphosphate (SA-PIP_2_) by phospholipase C. Since the spatially averaged PIP_2_ concentration at the IPM is ca. 1 mol% (44, 49, 50), we measured the effect of adding 1 mol% SAG to the IPM-mimetic GUVs on membrane binding of WCR-YDA. Figure 2B shows that GUV binding of WCR-YDA is only minimally affected by the presence of SAG. These results show that binding of WCR-YDA is not appreciably affected by the lipid environmental changes in the IPM.

We also measured the effect of the cytosolic extract of HeLa cells on membrane binding of WCR-YDA to determine if the cytosolic proteins can interfere with its IPM binding in HeLa cells. As shown in Fig. 2C, D, binding of WCR-YDA to IPM-mimetic GUVs was not affected by the extract of HeLa cells with the total protein concentration up to 12 mg/ml (i.e., the original HeLa cell extract with minimal dilution). Collectively, these results show that WCR-YDA is suited for in situ quantification of low-abundance cholesterol under pathophysiological conditions.

### Membrane binding properties of a non-D4-based ratiometric sensor

**Fig. 3:**
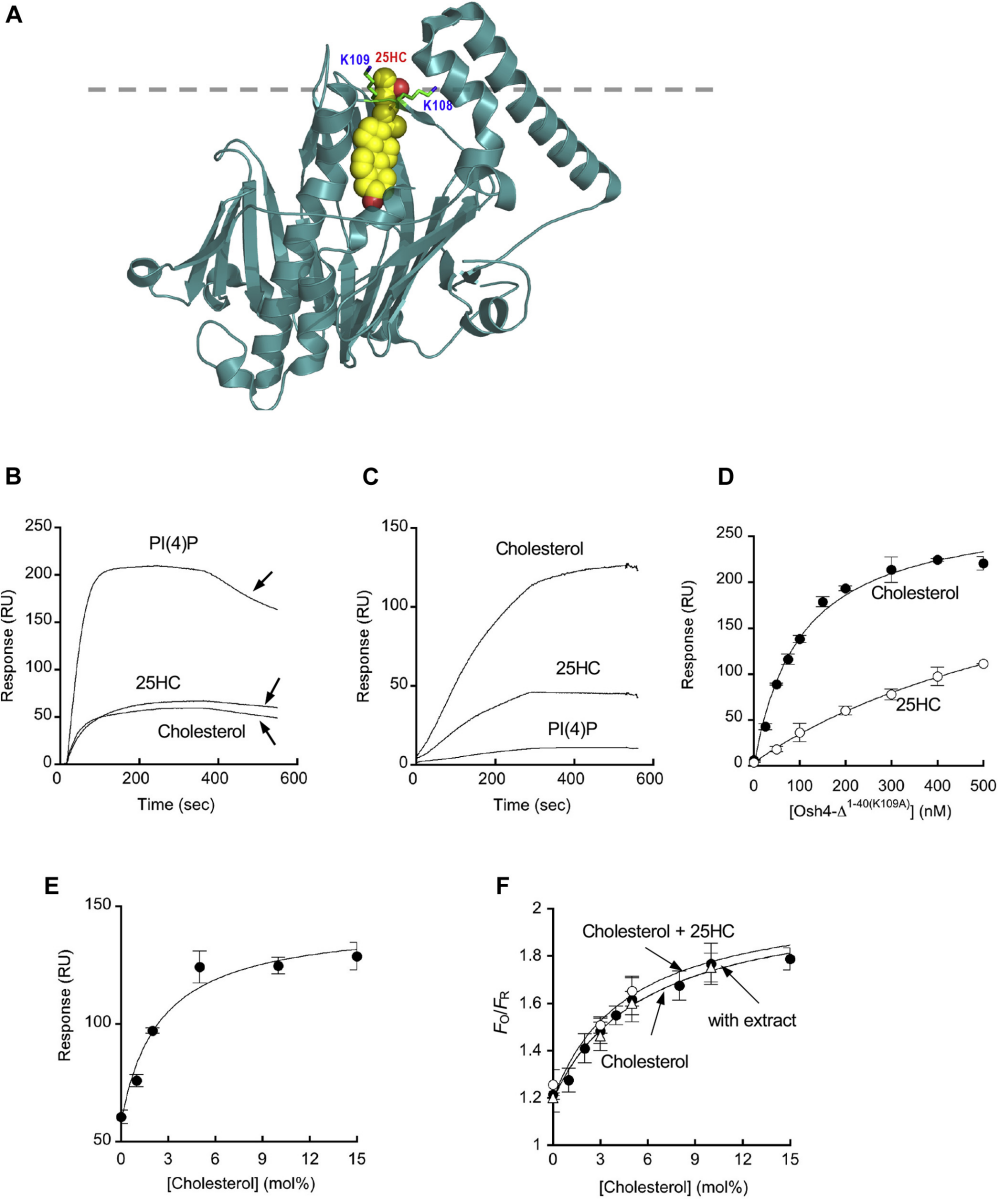
Structure and membrane binding properties of Osh4-derived proteins. A: The structure of Osh4 (Protein Data Bank ID: 1zhx) (in ribbon representation) with a bound 25HC molecule (space-filling representation) is shown with its membrane-binding surface pointing upward. N-terminal 40 residues forming the lid were removed for better illustration of 25HC-Osh4 interactions. The 25-hydroxyl group (red) is located close to K108. K109 that was mutated to Cys for fluorophore labeling is also shown. The gray line indicates the putative membrane surface. B: SPR sensorgrams for Osh4 binding to POPC/POPS/cholesterol (75/20/5), POPC/POPS/25HC (75/20/5), and POPC/POPS/PI(4)P (79/20/1) LUVs coated onto the L1 sensor chips. The sensorgrams were background corrected against that for POPC/POPS (80:20) vesicles. Only association phases are shown. Notice that binding kinetics show drops in SPR signals during membrane association (arrows), suggesting the lipid transfer activity during the process. C: SPR sensorgrams for Osh4-△^1-40(K109A)^ binding to the same vesicles described in (B). D: Determination of *K*_d_ for Osh4-△^1-40(K109A)^ binding to POPC/POPS/cholesterol (or 25HC) (77:20:5) LUVs by SPR analysis. The protein concentration was varied from 0 to 500 nM. *K*_d_ values determined for POPC/POPS/cholesterol (77:20:5) (closed circles) and POPC/POPS/25HC (77:20:5) (open circles) were 100 ± 16 nM and 800 ± 130 nM, respectively. E: Cholesterol-dependent vesicle binding of Osh4-△^1-40(K109A)^ determined by SPR analysis. The lipid composition of LUVs was POPC/POPS/cholesterol (70-*x*/20/*x*: *x* = 0–20 mol%). The binding curve was analyzed by nonlinear least squares analysis using a modified Langmuir equation: *y* = *y*_min_ + (*y*_max_ − *y*_min_)/(1 + *K*_d_/*x*) where *K*_d_, *y*_max_, and *y*_min_ are the analyte concentration (*x*) yielding half maximal binding, the maximal and minimal *y* values. Half maximal vesicle binding was achieved with 2.5 ± 1.1 mol% cholesterol. F: The GUV calibration curve of WCR-*e*Osh4 determined by fluorescence microscopy. Lipid compositions of GUVs were POPC/POPE/POPS/PI/cholesterol/PIP_2_ (20/50-x/20/9/x/1: *x* = 0–20 mol%) (close circles) and POPC/POPE/POPS/PI/cholesterol/PIP_2_/25HC (19/50-x/20/9/x/1/1: *x* = 0–20 mol%) (open circles). Cholesterol-dependent GUV binding was also measured in the presence of 2 mg/ml HeLa cell extract (open triangles). Half maximal membrane binding (see panel E for analysis) was achieved with 4.8 ± 0.8 mol% cholesterol, which was not altered by the presence of 25HC or the cell extract. Notice that simplified IPM-mimetic vesicles were used for all SPR measurements. Each data point represents the average ± SD from >3 independent measurements for (B) (*n* = 3), (C) (*n* = 3), and (D) (*n* = 5).

To preclude any possibility that quantification of [Chol]_*i*_ was compromised by intrinsic membrane binding properties of the D4 domain, we also developed a new ratiometric sensor from a protein unrelated to the D4 domain. Osh4 is a yeast cytosolic sterol transfer protein that is known to bind cholesterol as well as 25HC and phosphatidylinositol-4-phosphate (PI(4)P) (51) (Fig. 3A) and transfer sterols in a PI(4)P-dependent manner (52). Our SPR analysis showed that Osh4 has a much higher binding affinity for PI(4)P than cholesterol and 25HC (Fig. 3B). Its SPR sensorgrams showed a 4-fold larger binding signal for POPC/POPS/PI(4)P (79:20:1) LUVs coated on the sensor chip than those for POPC/POPS/cholesterol (or 25HC) (75:20:5) (Fig. 3B). Also, the sensorgrams exhibited protein dissociation during the protein association phase, which is uncommon among lipid-binding proteins (53, 54) but is consistent with its lipid-transfer activity. It was reported that removal of the N-terminal lid of Osh4 abrogates its sterol transfer activity and PI(4)P binding activity and that mutation of K109 suppresses 25HC binding (52), suggesting that a combination of N-terminal truncation and K109 mutation could convert Osh4 into a cholesterol-binding protein. To test this notion, we deleted the lid region (aa 1–40) and mutated K109 to Ala (Fig. 3A) and measured the membrane binding properties of the resulting mutant (Osh4-△^1-40(K109A)^). The SPR sensorgrams (Fig. 3C) demonstrate two striking effects of this structural alteration. First, Osh4-△^1-40(K109A)^ only had basal PI(4)P affinity and showed greatly enhanced selectivity for cholesterol over 25HC. Also, it did not show unusual binding kinetics observed with Osh4 WT and behaved like a typical lipid-binding domain (53, 54). We then determined the *K*_d_ values of Osh4-△^1-40(K109A)^ for POPC/POPS/cholesterol (75:20:5) (*K*_d_ = 100 ± 16 nM) and POPC/POPS/25HC (75:20:5) (*K*_d_ = 800 ± 130 nM) LUVs by measuring the SPR signals as a function of the protein concentration (Fig. 3D). For comparison, the *K*_d_ value determined for YDA-POPC/POPS/cholesterol (75:20:5) binding under the same conditions was 190 ± 25 nM. These *K*_d_ values thus verified the high affinity and specificity of Osh4-△^1-40(K109A)^ for cholesterol.

We also measured the binding of Osh4-△^1-40(K109A)^ to LUVs as a function of the cholesterol concentration in the vesicles (i.e., POPC/POPS/cholesterol (77-*x*:20:*x*: *x* = 0–20 mol%)). The plot of the maximal SPR signal versus the cholesterol content showed a typical Langmuir-type saturation pattern (Fig. 3E), which is in stark contrast to that of the D4 domain, which has a sigmoidal shape (supplemental Fig. S1). Of most importance, Osh4-△^1-40(K109A)^ was able to avidly bind POPC/POPS/cholesterol (77-*x*:20:*x*) LUVs even when its cholesterol content was below 5 mol% (Fig. 3E). Collectively, these results show that Osh4-△^1-40(K109A)^ can bind cholesterol molecules with high affinity and specificity, establishing Osh4-△^1-40(K109A)^ as an excellent template for a ratiometric cholesterol sensor.

We then replaced three endogenous cysteines of Osh4-△^1-40(K109A)^ to Ser (C68S/C98S/C229S) and introduced a cysteine in its membrane-binding surface (see Fig. 3A) as a single fluorophore labeling site (K108C) to yield *e*Osh4 (Osh4-△^1-40(C68S/C98S/K108C/K109A/C229S)^). Chemical labeling of *e*Osh4 with WCR produced a new ratiometric cholesterol sensor, WCR-*e*Osh4, which was then calibrated by fluorescence microscopy using IPM-mimetic GUVs with varying cholesterol concentrations (Fig. 3F; see also supplemental Fig. S2). As was the case with its parent molecule, Osh4-△^1-40(K109A)^ (see Fig. 3E), WCR-*e*Osh4 followed a Langmuir-type saturation pattern in cholesterol-dependent GUV binding (Fig. 3F), with a linear range covering 0–10 mol% cholesterol and the dynamic range comparable with that of WCR-YDA (see Fig. 2A). This calibration curve shows that WCR-*e*Osh4 is suited for ratiometric quantification of low-abundance cholesterol, as well as WCR-YDA. Also, the calibration curve was minimally altered by the presence of 1 mol% 25HC in GUV and 2 mg/ml HeLa cell extract (Fig. 3F), indicating that the potential presence of 25HC in the IPM or cytosolic proteins would not interfere with cholesterol binding of WCR-*e*Osh4.

### Quantification of IPM cholesterol in various mammalian cells

**Fig. 4:**
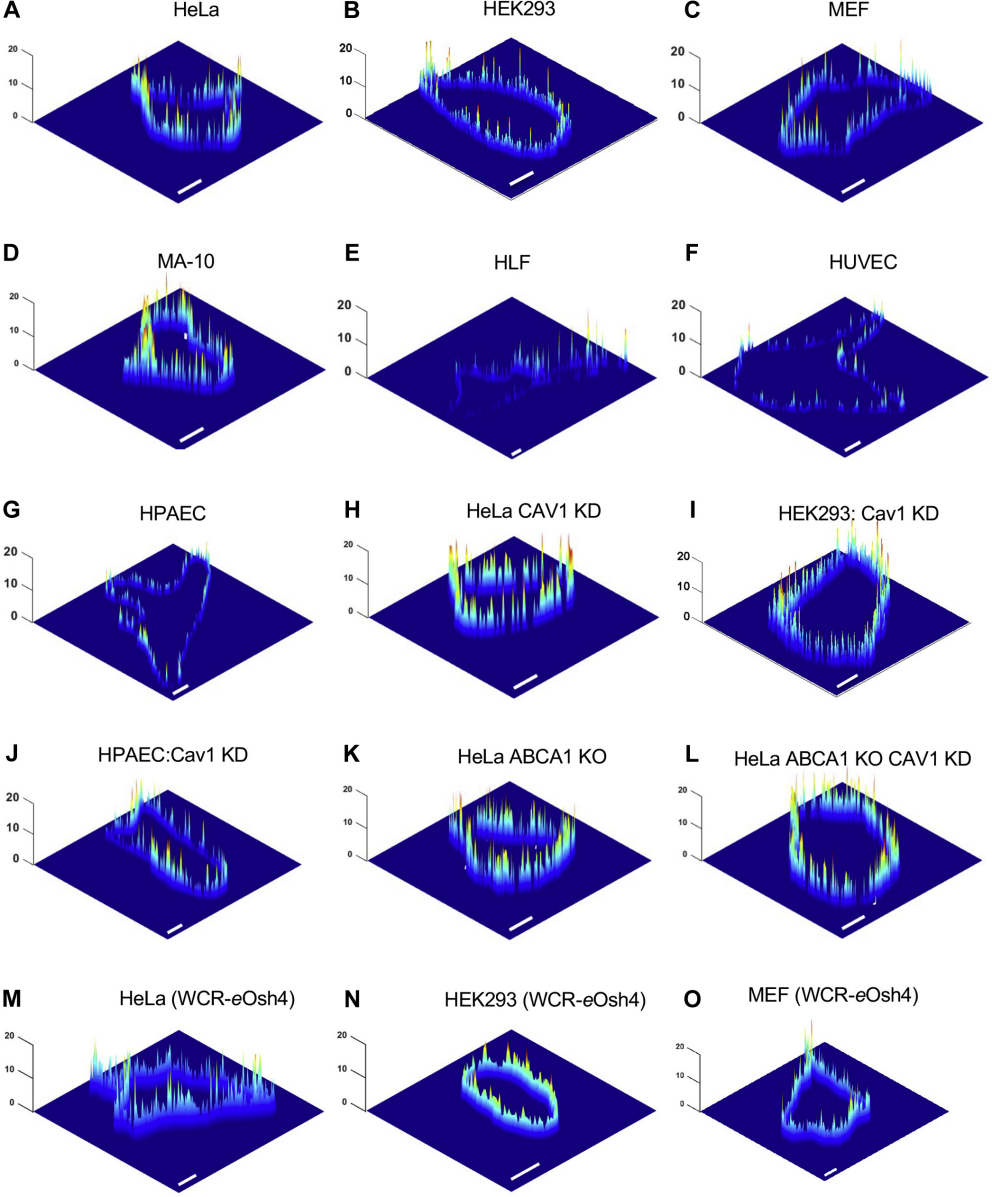
Spatially resolved IPM cholesterol concentration ([Chol]_*i*_) profiles calculated from two-channel cross-sectional images of various mammalian cells at a given time. A–L were obtained with microinjected WCR-YDA and (M–O) with microinjected WCR-*e*Osh4, respectively. Each cell is a representative of more than 30 cells analyzed. Spatially averaged [Chol]_*i*_ values for each cell type are summarized in Table 1. The *z*-axis scale indicates [Chol]_*i*_ in mol%. A pseudocoloring scheme with red and blue representing the highest (20 mol%) and the lowest (0 mol%) concentration, respectively, is used to illustrate the spatial [Chol]_*i*_ heterogeneity. Scale bars indicate 10 μm.

We previously reported that the average [Chol]_*i*_ values in common immortalized cell lines, including HeLa and HEK293 cells, vary from 2.1 to 3.4 mol% (19). To see if these low [Chol]_*i*_ values represent a universal trend in all mammalian cells, we extended our quantification to a wide variety of cells. We first redetermined [Chol]_*i*_ for HeLa and HEK293 cells using microinjected WCR-YDA. Three-dimensional [Chol]_*i*_ profiles for HeLa (Fig. 4A) and HEK293 cells (Fig. 4B) showed typical lateral heterogeneity within the IPM, and their average [Chol]_*i*_ values (3.6 ± 0.3 and 2.1 ± 0.3 mol% for HeLa and HEK293 cells, respectively) (Table 1) were essentially identical to those determined with NR3-YDA (19). Having established that WCR-YDA and NR3-YDA are functionally identical in in situ quantification of cholesterol, we then expanded our [Chol]_*i*_ determination with WCR-YDA to two additional cell lines and three primary cells. In immortalized mouse embryonic fibroblasts (MEFs), IPM cholesterol distribution showed similar lateral heterogeneity (Fig. 4C), and the average [Chol]_*i*_ (2.2 ± 0.5 mol%) (Table 1) lay within the reported range (19). We then determined [Chol]_*i*_ in MA-10 cells derived from the mouse Leydig cell tumor. MA-10 cells are a steroidogenic cell line that utilizes cholesterol in PM for steroid synthesis in mitochondria in a hormone-induced manner (55). Based on the clear PM localization of genetically encoded mCherry-D4 WT in MA-10 cells, it was reported that MA-10 cells might have a high level of cholesterol at the PM (55). Consistent with this report, MA-10 cells showed the highest basal level of IPM cholesterol among mammalian cells tested to date (Fig. 4D), with their average [Chol]_*i*_ reaching 8.1 ± 1.0 mol%, which is almost four times higher than that found in other cell lines (Table 1). However, this value is still much lower than the average concentrations of cholesterol at OPM in various mammalian cells (see Table 1) (19).

**Table 1:** Spatially averaged basal cholesterol concentrations at the plasma membranes of mammalian cells^a^

Cell Status	[Chol]_*i*_ (mol%)^b^							[Chol]_*o*_ (mol%)^c^	
	Cell Lines				Primary Cells			Cell Lines	Primary Cells
	HeLa	HEK293	MEF	MA-10	HPAEC	HUVEC	HLF	HeLa	HPAEC
WT^d^	3.6 ± 0.3	2.4 ± 0.3	2.2 ± 0.5	8.1 ± 1.0	0.6 ± 0.3	0.5 ± 0.2	0.6 ± 0.4	44.0 ± 4.6	49.0 ± 3.3
WT^d^ (WCR-*e*Osh4)	3.8 ± 0.4	2.4 ± 0.5	2.1 ± 0.5	NM^g^	NM	NM	NM	-	-
Cav1 KD^e^	6.9 ± 0.6	6.9 ± 0.7	7.2 ± 0.6	NM	2.0 ± 0.5	NM	NM	37.5 ± 4.5	43.0 ± 6.5
ABCA1-KO^f^	6.4 ± 0.4	NM	NM	NM	NM	NM	NM	NM	NM
ABCA1-KO/Cav1 KD	8.6 ± 0.6^c^	NM	NM	NM	NM	NM	NM	NM	NM

a Average ± SD from >3 independent determinations (for each determination, *n* > 30).

b IPM cholesterol concentration. All data were collected with WCR-YDA unless indicated otherwise (i.e., with WCR-*e*Osh4).

c OPM cholesterol concentration. All data were collected with DAN-D4.

d Wild type. [Chol]_*i*_ quantification was separately performed with WCR-YDA and WCR-*e*Osh4, respectively. *P* values between two measurements were 0.59, 1.00, and 0.66 for HeLa, HEK293, and MEF cells, respectively.

e siRNA-mediated gene knockdown.

f Gene knockout.

g Not measured.

Spatiotemporally resolved cholesterol concentrations in primary cells have not been reported. Our previous study found a good correlation between [Chol]_*i*_ and cell growth and proliferation activity (19). This in turn suggested that primary cells might have lower [Chol]_*i*_ values than transformed cell lines. To test this notion, we quantified [Chol]_*i*_ in three different primary cells, HLFs, HUVECs, and HPAECs. Three-dimensional [Chol]_*i*_ profiles in these cells consistently showed extremely low abundance of IPM cholesterol (Fig. 4E–G). As summarized in Table 1, the average [Chol]_*i*_ values in these primary cells are less than one-third of those calculated for common immortalized cell lines. This new finding lends further credence to the notion that [Chol]_*i*_ is maintained low under physiological conditions. Of interest, [Chol]_*o*_ in HPAEC was considerably higher (i.e., 11% increase; *P* = 0.01) than that in HeLa cells (Table 1), suggesting that IPM cholesterol is further shuttled to the OPM in primary cells than in transformed cell lines.

We also determined [Chol]_*i*_ using WCR-*e*Osh4 microinjected into cells. Three-dimensional [Chol]_*i*_ profiles for HeLa (Fig. 4M), HEK293 (Fig. 4N), and MEF cells (Fig. 4O) showed the same lateral heterogeneity within the IPM as observed with WCR-YDA for these cells (Fig. 4A–C). The spatially averaged [Chol]_*i*_ value for each cell line determined with WCR-*e*Osh4 is statistically indistinguishable (i.e., *P* > 0.5) from that determined with WCR-YDA (Table 1). Taking into account that WCR-YDA and WCR-*e*Osh4 derived from two distinctively different proteins with totally different origins, structures, and membrane binding properties, this remarkable consistency in [Chol]_*i*_ values provides compelling evidence for the validity of our [Chol]_*i*_ values.

### Validation of the IPM cholesterol concentrations using EGFP-D4 domain proteins

To validate our [Chol]_*i*_ values by nonratiometric analysis, we quantitatively analyzed the PM localization patterns of genetically encoded enhanced green FP (EGFP)-tagged D4 WT, D434A, and YDA in those cells used for our ratiometric imaging. FP-D4 domain constructs have been extensively used for probing intracellular cholesterol in various mammalian cells but have often produced conflicting results (34, 36, 37, 56), partly because the expression levels of these proteins have not been properly controlled. It has been long known that overexpression of proteins in mammalian cells causes a wide range of nonphysiological effects, including abnormal subcellular localization. To investigate the relationship between the expression level of exogenous EGFP-D4 proteins and their PM localization behaviors, we first devised a robust protocol to selectively analyze cells with a defined level of D4 protein expression. Since fine tuning of the protein expression level is technically challenging, we first carefully adjusted the transfection level and expression time of each of EGFP-D4 domains to enrich those cells expressing high and low levels of the protein, respectively. After confocal imaging of these enriched cells under the same conditions, we then selected only those cells that met our predefined expression criteria during the image analysis. Our control experiments using the free EGFP solution showed a good linear correlation between the total fluorescence intensity per area and the EGFP concentration (supplemental Fig. S3). This standard plot was used to estimate the cellular concentration of an EGFP-D4 domain from the mean fluorescence intensity value of the cross-sectional image of a single cell. Using this calibration plot, we selected those individual cells whose cellular EGFP-D4 domain concentrations were comparable with or lower (i.e., ≤250 nM) than the typical cellular concentration of the microinjected WCR-YDA (or WCR-*e*Osh4) as low-expression cells. We also selected those cells whose expression level of EGFP-D4 domains is more than twice higher than this criterion (i.e., >500 nM) as high-expression cells.

**Fig. 5:**
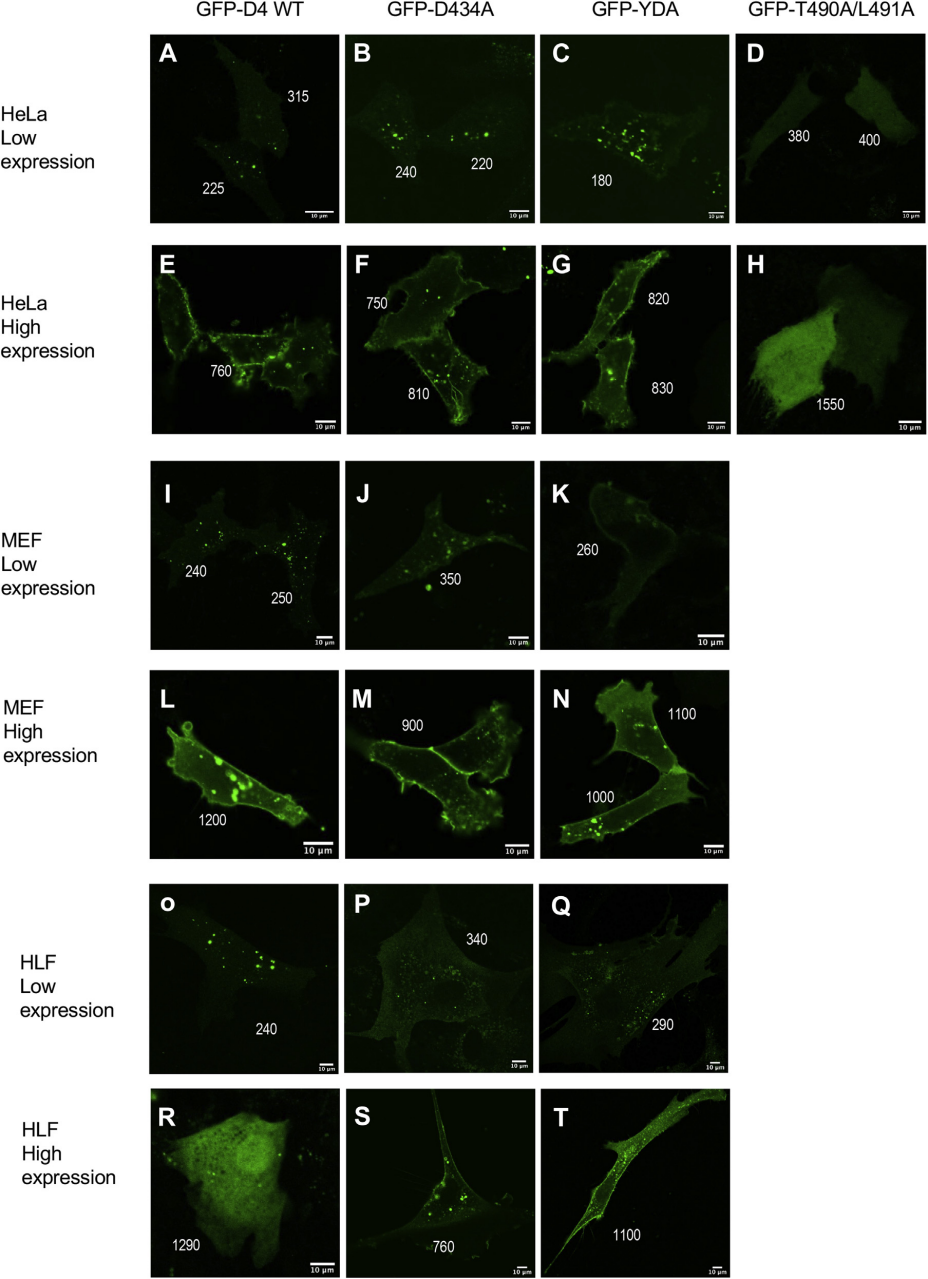
Subcellular localization of EGFP-tagged D4 domains in mammalian cells with the low IPM cholesterol concentration. Each cell is representative of >50 cells showing a similar pattern. Low-expression cells have D4 domains <250 nM, whereas high-expression cells contain >500 nM D4 domains. Scale bars indicate 10 μm. Numbers next to each cell indicate its mean optical density from which the expression level of an EGFP-D4 domain is estimated. A–D: low-expression HeLa cells; E–H: high-expression HeLa cells; I–K: low-expression MEF cells; L–M: high-expression MEF cells; O–Q: low-expression HLF cells; R–T: high-expression HLF cells.

First, we measured the subcellular localization of EGFP-D4-WT, -D434A, and -YDA under low- and high-expression conditions in those cells with low [Chol]_*i*_. In low-expression HeLa cells, EGFP-D4-WT (Fig. 5A) and EGFP-D4-D434A (Fig. 5B) were not detectable in PM and only EGFP-D4-YDA showed faint PM localization (Fig. 5C). These low-intensity cell images were not due to autofluorescence of cells as untransfected HeLa cells produced no detectable fluorescence signal with our microscopy setting (see supplemental Fig. S4). In stark contrast, all three D4 domains (Fig. 5E–G) showed clear PM localization in high-expression HeLa cells. The IPM localization of the D4 domain WT (and mutants) was due to specific cholesterol binding because it was abrogated by the mutation of residues essential for cholesterol binding (T490A/L491A) (57) (Fig. 5D–H). A similar trend was observed with MEF cells. Low protein expression allowed only EGFP-D4-YDA to show a minor degree of PM localization (Fig. 5I–K), whereas high expression drove all D4 domains to the PM (Fig. 5L–N). Finally, in primary HLF cells with the lowest [Chol]_*i*_ value (i.e., 0.6 mol%), no D4 domain, including EGFP-D4-YDA, showed appreciable PM localization when their expression was kept low (Fig. 5O–Q). Even in these cells, however, EGFP-D4-D434A (Fig. 5S) and -YDA (Fig. 5T) displayed moderate to strong PM localization, respectively, under high expression conditions. EGFP-D4-WT showed no PM localization under the same conditions (Fig. 5R). Although low and high protein expression levels were only arbitrarily defined in this study based on the average concentration of our microinjected IPM cholesterol sensors, these results clearly illustrate how greatly the expression level of D4 domains dictates the degree of their membrane translocation.

**Fig. 6:**
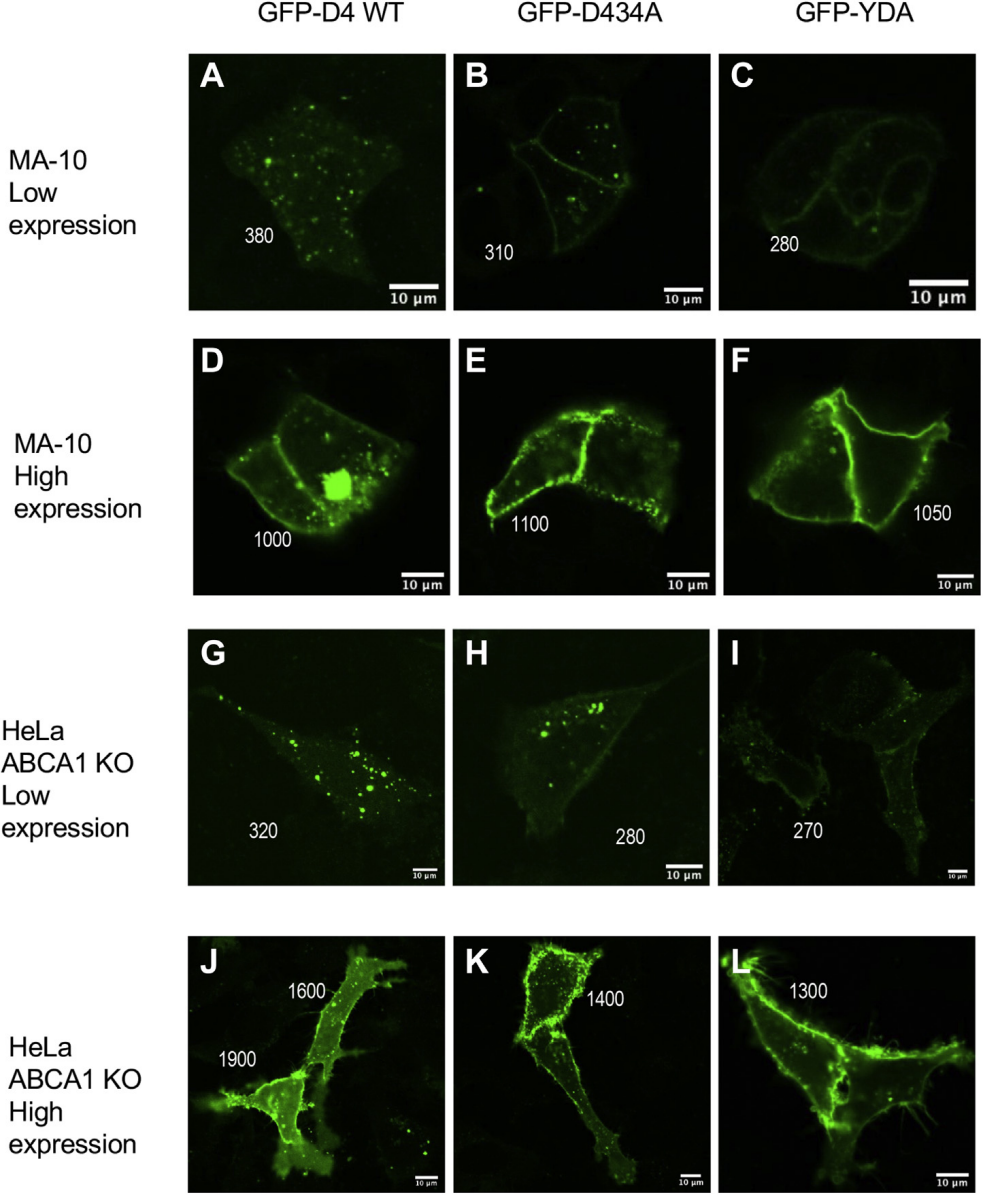
Subcellular localization of EGFP-tagged D4 domains in mammalian cells with the relatively high IPM cholesterol concentration. Low-expression cells have D4 domains <250 nM, whereas high-expression cells contain >500 nM D4 domains. Scale bars indicate 10 μm. Numbers next to each cell indicate its mean optical density from which the expression level of an EGFP-D4 domain is estimated. A–C: low-expression MA-10 cells; D–F: high-expression MA-10 cells; G–I: low-expression ABCA1-null HeLa cells; J–L: high-expression ABCA1-null HeLa cells.

We also measured the PM localization of EGFP-D4 domains in cells with higher [Chol]_*i*_ values. In MA-10 cells, EGFP-D4-D434A (Fig. 6B) and EGFP-D4-YDA (Fig. 6C) showed considerable PM localization even in low-expression cells. However, EGFP-D4-WT was not localized at the IPM under these conditions (Fig. 6A). All D4 domains were enriched at PM in high-expression MA-10 cells (Fig. 6D–F). A similar trend was observed in ABCA1-deficient HeLa cells in which [Chol]_*i*_ is elevated to 6.4 mol% (see Table 1 and Fig. 4K). That is, EGFP-D4-D434A and EGFP-D4-YDA were found at PM to some degree in low-expression cells (Fig. 6G–I), whereas all D4 domains were predominantly localized to PM in high-expression cells (Fig. 6J–L).

**Fig. 7:**
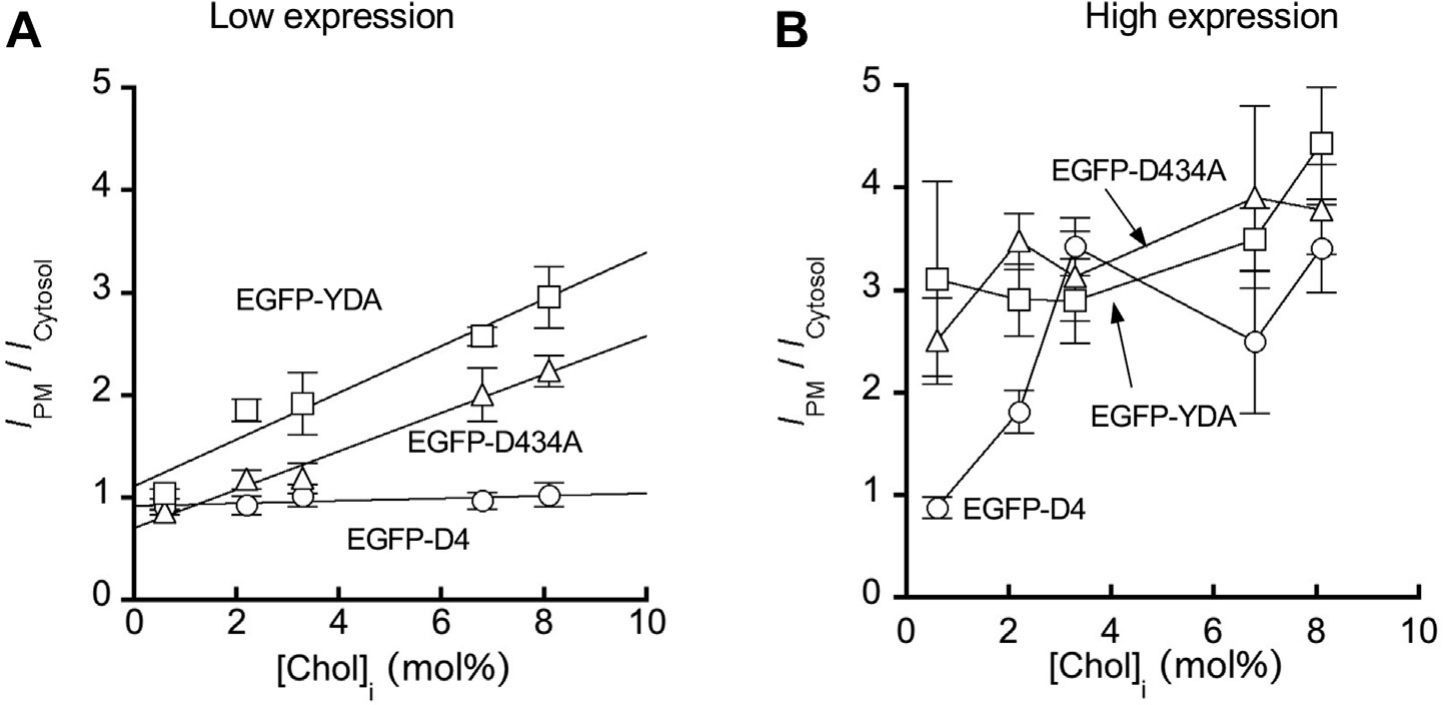
Correlation between the IPM cholesterol concentration and the degree of IPM localization of EGFP-D4 domains in low- (A) and high- (B) expression cells. [Chol]_*i*_ values for five different cells were taken from Table 1. The degree of IPM localization (*I*_PM_/*I*_Cytosol_) of EGFP-D4 WT (open circles), -D434A (open triangles), and -YDA (open squares) were calculated from Figs. 5 and 6. *I*_PM_/*I*_Cytosol_ (ratio of fluorescence intensity at PM to that in the cytosol) for each cell type was calculated by the fluorescence intensity line profile analysis from >10 cells. Linear regression was used to fit the plots in (A), but line connection was used for (B) owing to a poor linear correlation.

To quantitatively determine the correlation between [Chol]_*i*_ and the degree of IPM localization of EGFP-D4 domains, we plotted the ratio of the average fluorescence intensity at PM to that in the cytosol (*I*_PM_/*I*_Cytosol_) as a function of [Chol]_*i*_ in various cells for each of three EGFP-D4 domains. As shown in Fig. 7A, in low EGFP-D4-YDA- and EGFP-D4-D434A-expressing cells, an excellent linear correlation was observed between [Chol]_*i*_ and *I*_PM_/*I*_Cytosol_. However, no such linearity was observed for any EGFP-D4 domains in high-expression cells (Fig. 7B). Overall, when the expression level of EGFP-tagged D4 domains was kept close to the cellular concentration of microinjected WCR-YDA, the degree of their PM localization in different cells showed an excellent quantitative correlation with [Chol]_*i*_ for these cells determined by our ratiometric imaging. However, uncontrolled overexpression of these proteins drove their PM localization, yielding misleading results.

### Availability of IPM cholesterol is controlled by cholesterol-sequestering proteins

Our [Chol]_*i*_ values represent the concentrations of cholesterol readily available to the ratiometric sensors present in the cytosol. Consistently low [Chol]_*i*_ values, particularly in primary cells, raise a possibility that there might be sequestered pools of IPM cholesterol that are not available to cytosolic protein sensors. The presence of sequestered lipid pools at the IPM whose availability is controlled by a buffering protein has been proposed for signaling lipids, most notably PIP_2_ (58). Numerous membrane proteins in the PM have been reported to bind cholesterol (9). It is therefore conceivable that a significant proportion of hydrophobic cholesterol molecules are locally sequestered by these membrane proteins. As a proof of concept, we selected one representative high-affinity PM-resident cholesterol-binding protein, caveolin-1 (Cav1) (59), and measured the effect of suppressing its expression on IPM cholesterol availability. Caveolins (Cav1, Cav2, and Cav3) are integral membrane proteins essential for the formation and maintenance of caveolae (60, 61), PM invaginations that have been reported to be rich in cholesterol (61, 62, 63). Cav1 knockdown by siRNA greatly increased [Chol]_*i*_ to ca. 7 mol% in HeLa (Fig. 4H), HEK293 (Fig. 4I), and MEF (Table 1) cells. Even for primary HPAECs with an extremely low basal IPM cholesterol level, Cav1 knockdown caused a large 3.3-fold increase in [Chol]_*i*_ from 0.6 to 2.0 mol% (Fig. 4J and Table 1). These results suggest that caveolae contain a significant pool of Cav1-sequestered cholesterol that are not available to WCR-YDA. Of interest, Cav1 knockdown did not increase [Chol]_*o*_ in HeLa and HPAEC cells (Table 1), suggesting that cholesterol sequestration by Cav1 is mostly limited to IPM cholesterol. A modest decrease in [Chol]_*o*_ may be due to partial suppression of the floppase activity of ABCA1 by the Cav1 knockdown (see below).

Cav1 has also been implicated in assisting ABCA1-mediated cholesterol efflux from the peripheral cells (64, 65), although the exact mechanism underlying this observation remains unknown. Since ABCA1 plays a crucial role in shuttling IPM cholesterol to OPM (19), this suggests that Cav1 knockdown may increase [Chol]_*i*_ (and decrease [Chol]_*o*_; see above) by suppressing the floppase activity of ABCA1. To sort out the effects of Cav1 knockdown on disruption of floppase activity of ABCA1 and the release of caveolae-sequestered IPM cholesterol, we thus measured the effect of Cav1 knockdown on ABCA1-deficient HeLa cells. As shown in Fig. 4K, ABCA1-deficient HeLa cells already have a high average [Chol]_*i*_ (see Table 1). Even for these cells, Cav1 knockdown further enhanced [Chol]_*i*_ to a significant degree (*P* <0.0001) (see Table 1 and Fig. 4L). Collectively, these results support the notion that Cav1 sequesters a significant portion of IPM cholesterol in caveolae in all mammalian cells tested in this study. Taking into account the potential involvement of other PM proteins in cholesterol sequestration, the total IPM cholesterol concentrations in mammalian cells might thus be considerably higher than the available IPM cholesterol concentrations (i.e., [Chol]_*i*_) determined by our ratiometric sensors.

## Discussion

Asymmetric distribution of lipids in the two leaflets of PM (and other cell membranes) of mammalian cells has been well established (21, 66, 67). For example, sphingomyelin is localized predominantly at the OPM, whereas aminophospholipids and phosphoinositides are primarily found at the IPM. A recent study also showed transbilayer asymmetry of phospholipid unsaturation across PM, with 2-fold higher abundance of unsaturated phospholipids at IPM (68). However, the transbilayer distribution of cholesterol in the PM remains controversial (21). An earlier notion that cholesterol is evenly distributed between the two leaflets is mainly based on the findings that it could rapidly flip-flop between the lipid bilayer (69, 70, 71), but the fast flip-flop of cholesterol has been experimentally disputed (72). Recent studies have deepened the controversy as some laboratories reported cholesterol enrichment at IPM (23, 24), whereas our quantitative imaging showed that the cholesterol concentration at IPM is much lower than that at OPM (19, 20). The present study provides new experimental data that help resolve both technical and conceptual aspects of the controversy.

Most of the debate on the transbilayer asymmetry of PM cholesterol has focused on the technical aspect. Specifically, our reported [Chol]_*i*_ values have been challenged on the grounds that D4 domain-derived proteins cannot be used for IPM cholesterol quantification owing to their unique membrane binding properties (22, 56). PFO and other bacterial toxins have evolved to function optimally at the OPM of mammalian cells with high [Chol]_*o*_ and consequently cannot readily interact with membranes containing low levels of cholesterol (35, 73, 74). This phenomenon has been explained by different models, including the stochiometric phospholipid-cholesterol complex model (43, 75) and the molecular umbrella model (76, 77). Our previous (19) and current results show that we have overcome these technical limitations by converting the PFO-D4 domain through protein engineering and chemical modification into ratiometric sensors (NR3-YDA and WCR-YDA) that possess a unique ability to effectively interact with membranes containing low concentrations of cholesterol under physiological conditions. That is, they have much higher cholesterol affinity than D4 WT, an extended linear response range covering 0–15 mol% of cholesterol concentration without a threshold, and insensitivity to the lipid environment and the presence of cytosolic proteins under physiological conditions.

The mechanisms by which soluble proteins, such as the D4 domain or PDZ domains (14, 15), interact with hydrophobic cholesterol in the lipid bilayer are not fully understood (40). Deducing from the membrane binding mechanism of C1 domains that interact with another hydrophobic lipid, diacylglycerol (54, 78), one would expect that cholesterol binding of soluble proteins involves partial membrane penetration of proteins into the hydrophobic core of the lipid bilayer. We previously showed that aromatic residues, most notably Trp, on the membrane-binding surface of a protein greatly facilitate membrane penetration of the protein (54, 79). Thus, introduction of two extra Trp residues to the membrane-binding surface of the D4 domain as well as chemical conjugation of an aromatic fluorophore to the same region should dramatically enhance the ability of WCR-YDA (and NR3-YDA) to penetrate the membrane and interact with cholesterol molecules located in the hydrophobic region of the lipid bilayer.

Unlike the D4 domain and derivatives, Osh4 is a cytosolic sterol transfer protein whose interaction with IPM cholesterol is intrinsic to its function (51, 52). Consequently, *e*Osh4, which was engineered from Osh4 to remove the sterol transfer activity and the affinity for 25HC and PI(4)P, and the *e*Osh4-derived ratiometric sensor (WCR-*e*Osh4) efficiently and tightly bind vesicles containing low concentrations of cholesterol (i.e. ≤5 mol%). WCR-*e*Osh4 has high affinity and specificity for cholesterol-containing membranes and can effectively interact with low-abundance cholesterol in the membrane, independent of its lipid environment. Taken together, WCR-YDA and WCR-*e*Osh4 are well suited for in situ quantification of IPM cholesterol. Of most importance, WCR-*e*Osh4 and WCR-YDA produced essentially the same [Chol]_*i*_ values in three different cells. Considering that WCR-YDA and WCR-*e*Osh4 derive from two completely unrelated proteins with distinctively different origins, structures, and properties, these results demonstrate that our low [Chol]_*i*_ values represent genuine available IPM cholesterol concentrations and are not attributed to intrinsic problems associated with the D4 domain-based sensors (56).

Furthermore, our rigorous imaging analyses of EGFP-D4 WT, D434A, and YDA demonstrate that, when cells with a defined level of protein expression are selectively analyzed, the degree of their PM localization in different mammalian cells is quantitatively correlated with [Chol]_*i*_ of the cells determined by our ratiometric sensors. These results thus validate not only the utility of our engineered cholesterol sensors but also our in vitro calibration-based ratiometric quantification approach. It should be noted that, under the uncontrolled overexpression conditions, all cholesterol probes are indiscriminately driven to the IPM in all these cells except primary cells with extremely low [Chol]_*i*_. We thus caution against using FP-tagged D4 domains as intracellular cholesterol probes without rigorous controls (56).

The cholesterol concentration at the PM, whether IPM or OPM, in primary cells has never been reported. Our quantification in primary fibroblasts and endothelial and epithelial cells shows that [Chol]_*i*_ is four to six times lower in primary cells than in immortalized cell lines. These exceptionally low [Chol]_*i*_ values in primary cells support the physiological relevance and significance of our previous findings that [Chol]_*i*_ is kept low in unstimulated cells because of its role in cell growth and proliferation (15, 16, 19, 20).

The current study also provides new insight into the conceptual aspect of the transbilayer asymmetry of PM cholesterol in terms of total versus available cholesterol concentrations. As described above, our [Chol]_*i*_ values represent the IPM cholesterol concentrations available to cytosolic proteins, whether they are lipid sensors or signaling proteins, but not necessarily the total IPM cholesterol concentrations. This study demonstrates the presence of an extra pool of Cav1-sequestered IPM cholesterol that is unavailable to cytosolic ratiometric sensors. Cav1 is a high-affinity cholesterol-binding protein in the PM (59). In addition to its critical role in the formation and maintenance of caveolae (60, 61), Cav1 has been implicated in cellular cholesterol homeostasis, including cholesterol efflux from peripheral cells (64, 65). However, the exact role of Cav1 in cholesterol efflux remains controversial and little is known about whether OPM or IPM cholesterol is used for efflux. Some reports support that Cav1 facilitates cholesterol efflux by regulating ABCA1 and other transporters. For example, Cav1 overexpression increased cholesterol efflux in skin fibroblasts (80) and hepatic cells (81), whereas suppression of Cav1 expression reduced cholesterol efflux in human monocytic leukemia cell line THP-1 (82). In contrast, suppression of Cav1 expression enhanced cholesterol efflux in NIH 3T3 cells (83) and gene ablation of Cav1 had little effect on cholesterol efflux in MEFs (84, 85) and mouse peritoneal macrophages (84). These contradicting results indicate that the effect of Cav1 on cholesterol efflux is highly context dependent. Our finding that Cav1 knockdown consistently raises [Chol]_*i*_ to a large degree in a wide variety of cells, including ABCA1-KO HeLa cells, supports the notion that caveolae-sequestered IPM cholesterol is released upon Cav1 depletion. Caveolae contain three caveolin isoforms and other proteins, such as cavins, which are important for their structural integrity (60). Also, many PM-resident proteins have been reported to contain tightly bound cholesterol molecules (9, 10, 11, 12, 13). Thus, the level of unavailable IPM cholesterol might be even higher than estimated by the Cav1 suppression.

This potential gap between the available and total IPM cholesterol concentration values may partially account for the discrepancy among reported PM cholesterol concentrations determined by different methods (19, 25, 26, 27). It may also provide a partial answer to the question of energetics of transbilayer cholesterol asymmetry at the PM. Steck and Lange (22) argued that our reported cholesterol asymmetry is difficult to sustain by the ABCA1-mediated cholesterol shuttling owing to an estimated high energetical demand. If the actual ratio of total OPM cholesterol to total IPM cholesterol is lower than that calculated from our [Chol]_*i*_ values, the transbilayer cholesterol gradient may demand less energy to maintain. However, the real answer to this energetics question would require further mechanistic elucidation and quantitative assessment of IPM-to-OPM cholesterol translocation by ABCA1 and other cholesterol floppases.

Finally, it should be stressed that [Chol]_*i*_, our experimentally determined available IPM cholesterol concentration, is a physiologically relevant and important parameter in terms of cell signaling and regulation because our cholesterol sensors and cytosolic signaling proteins will experience the same degree of IPM cholesterol availability. Accumulating evidence from our group (14, 15, 16) and others (28, 29, 30) has supported the notion that IPM cholesterol is directly involved in cellular regulation and function via interaction with cytosolic proteins. Thus, cells might control the amplitude and duration of cholesterol-mediated signal transduction to cytosolic proteins by tightly regulating the availability of IPM cholesterol. We previously showed that agonist-dependent phosphorylation of ABCA1 inhibits its cholesterol transport activity and increases [Chol]_*i*_, which in turn stimulates canonical Wnt signaling (19). Our recent study also suggested that hedgehog signaling activity is regulated by the Patched1-mediated cholesterol transport, which controls [Chol]_*i*_ (20). The present study implies that regulation of IPM cholesterol availability by Cav1 and other membrane proteins might potentially serve as another control mechanism for [Chol]_*i*_ and IPM cholesterol-mediated cell signaling. It is possible that the cholesterol-sequestering activity of these proteins is reversibly controlled by, for example, posttranslational modification. Undoubtedly, further studies are required to fully investigate this potentially important mechanism.

## Data availability

All data are contained within the article. The raw data will be shared upon request: Contact Wonhwa Cho (University of Illinois at Chicago, Email: wcho@uic.du).

## Supplemental data

This article contains supplemental data.

## Conflict of interest

The authors declare that they have no conflicts of interest with the contents of this article.
