# Supplementary material for: Evaluation of the available cholesterol concentration in the inner leaflet of the plasma membrane of mammalian cells
Source: J Lipid Res. 2021 May 5;62:100084. doi: 10.1016/j.jlr.2021.100084 (PMC8178126; doi:10.1016/j.jlr.2021.100084)
Supplement: Supplemental Figures S1 to S4 [file mmc1.pdf]

SUPPLEMENTARY INFORMATION

Title: Evaluation of the accessible cholesterol concentration in the inner leaflet of the plasma membrane of mammalian cells.

Authors: Pawanthi Buwaneka[1,#], Arthur Ralko[1,#], Shu-Lin Liu[2], and Wonhwa Cho[1,*]

[1]*Department of Chemistry, University of Illinois at Chicago, Chicago, IL 60607, U.S.A.*
[2] *Present address: Department of Chemistry, Nankai University, Tianjin, China.*

[#]These authors equally contributed to this work
**Corresponding author: Wonhwa Cho, Department of Chemistry, University of Illinois at Chicago, Chicago, IL 60607, USA;* E-mail: wcho@uic.edu

Supplemental Fig. S1. Cholesterol dependency of vesicle binding of wild type (WT) D4 domain. Maximal RU values determined from the sensorgrams for binding of the WT D4 domain to POPC (or 1-stearoyl-2-arachidonoyl-*sn*-glycero-3-phosphocholine (SAPC))/POPS/cholesterol (70-*x*:30:*x*, *x* = 0-50 mol%) were plotted against the cholesterol concentration in the vesicles. Calibration curve fitting for WCR-YDA was performed by non-linear least-squares analysis using the equation: $y = y_{min} + (y_{max} - y_{min})$ (1 + Exp(($K_{1/2}$ − [Chol])/S). $K_{1/2}$, y_{max}, y_{min} , and S are [Chol] yielding half maximal binding (in mol%), the maximal and minimal y values and the Slope (or Stiffness) constant, respectively (1). Notice that simplified IPM-mimetic vesicles were used for the SPR analysis to cover a wide concentration range of cholesterol. Each data represents the average ± SD of triplicate measurements.

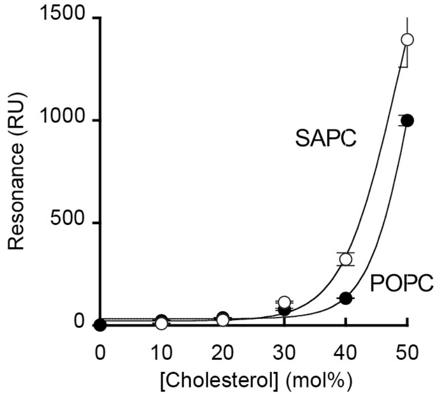

Supplemental Fig. S2. Two channel images of giant unilamellar vesicles (GUVs) interacting with cholesterol sensors.
For two different cholesterol sensors (WCR-YDA and WCR-*e*Osh4) interacting with GUVs (POPC/POPE/POPS/PI/cholesterol/PIP_2 (20/50-x/20/9/x/1) with 3 mol% and 10 mol% cholesterol, respectively, orange channel (derived from membrane-bound sensors) and red channel (derived from both free and membrane-bound sensors) images. The ratio of orange to red channel intensity at the membrane was calculated for each of >10 GUVs used for calibration at each cholesterol concentration. One representative vesicle image for each sensor at each cholesterol concentration was selected for illustration. Scale bars indicate 10 μm.

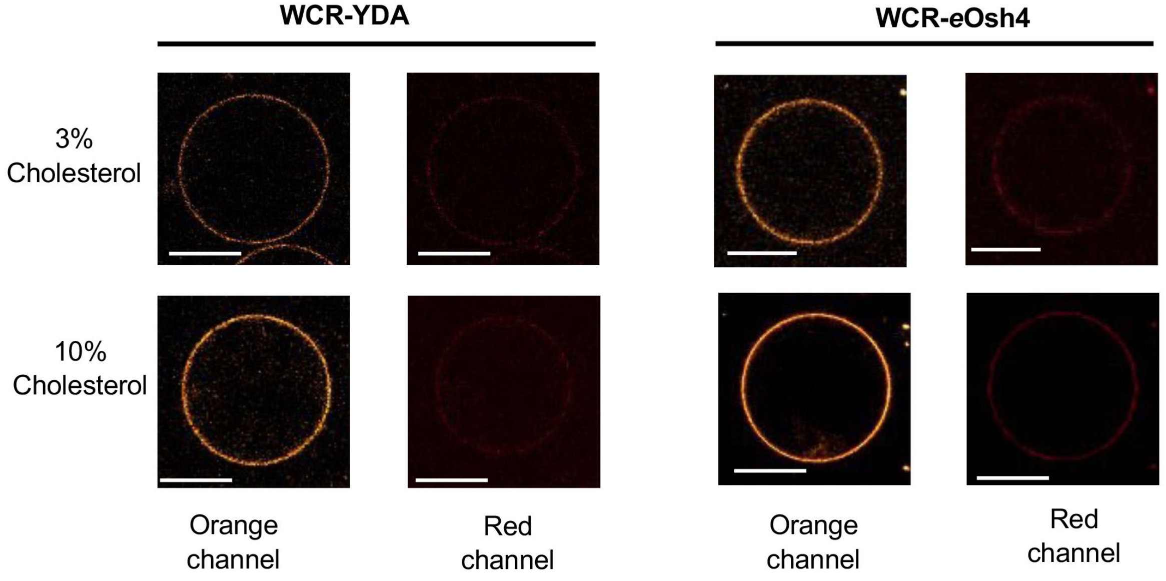

Supplemental Fig. S3. Correlation between the EGFP concentration and the fluorescence intensity per area.
Fluorescence intensity of each well containing different concentrations of EGFP in the phosphate buffer saline (pH 7.4) solution was measured using the FV3000 confocal microscope with the same setting employed for cellular imaging EGFP-D4 domains. In Image-Pro Plus, mean optical density (arbitrary unit (AU)/pixel) values were calculated for individual wells and plotted against the EGFP concentration to yield the calibration plot. The plot was analyzed by linear regression. Each data is the average ± S.D. of triplicate measurements.

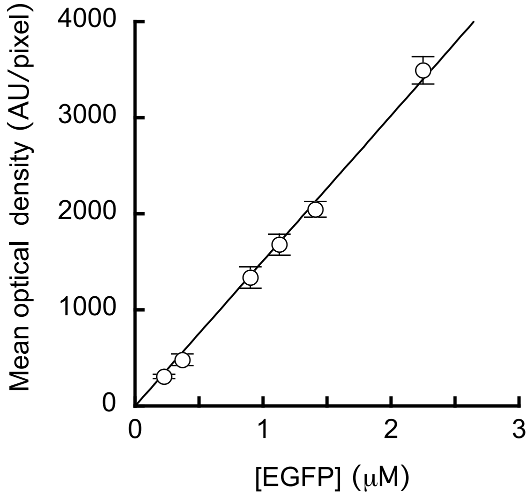

Supplemental Fig. S4. Fluorescence images of untransfected HeLa cells
Untransfected HeLa cells were imaged with the same microscope setting employed for imaging HeLa cells transfected with EGFP tagged D4 domains. Fluorescence images of cells (the left panel) show no detectable signal (mean optical density ≤ 2 in Image-Pro Plus). Differential interference contrast (DIC) images of the same cells were shown in the right panel to show the location of cells. Scale bars indicate 10 μm.

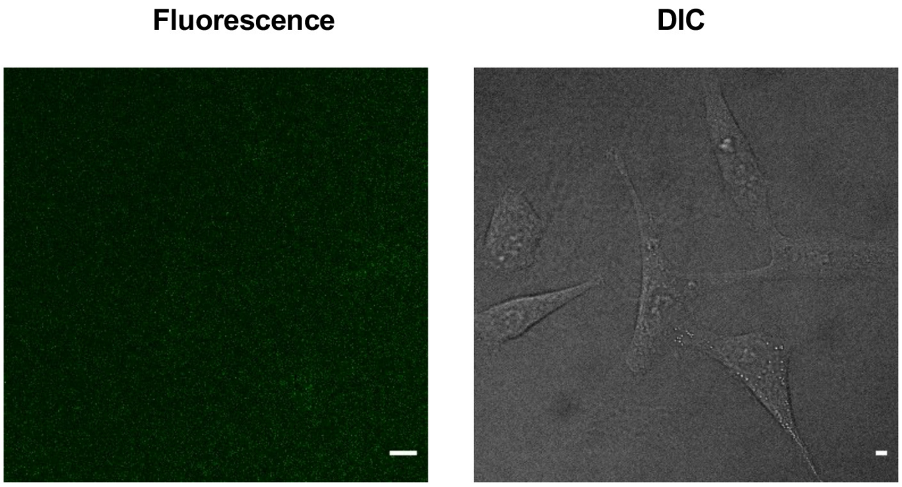

References

1. Liu, S. L., R. Sheng, J. H. Jung, L. Wang, E. Stec, M. J. O'Connor, S. Song, R. K. Bikkavilli, R. A. Winn, D. Lee, K. Baek, K. Ueda, I. Levitan, K. P. Kim, and W. Cho. 2017. Orthogonal lipid sensors identify transbilayer asymmetry of plasma membrane cholesterol. *Nat. Chem. Biol.* **13**: 268-274.
